# Protoporphyrin IX in serum of high-grade glioma patients: A novel target for disease monitoring via liquid biopsy

**DOI:** 10.1038/s41598-024-54478-y

**Published:** 2024-02-21

**Authors:** Anna Walke, Christopher Krone, Walter Stummer, Simone König, Eric Suero Molina

**Affiliations:** 1 Department of Neurosurgery, University Hospital of Münster, Albert-Schweitzer-Campus 1, A1, 48149 Münster, Germany; 2 Core Unit Proteomics, Interdisciplinary Centre for Clinical Research, University of Münster, Münster, Germany

**Keywords:** Tumour biomarkers, CNS cancer, Cancer screening, Diagnostic markers

## Abstract

High-grade gliomas (HGG) carry a dismal prognosis. Diagnosis comprises MRI followed by histopathological evaluation of tissue; no blood biomarker is available. Patients are subjected to serial MRIs and, if unclear, surgery for monitoring of tumor recurrence, which is laborious. MRI provides only limited diagnostic information regarding the differentiation of true tumor progression from therapy-associated side effects. 5-aminolevulinic acid (5-ALA) is routinely used for induction of protoporphyrin IX (PpIX) accumulation in malignant glioma tissue, enabling improved tumor visualization during fluorescence-guided resection (FGR). We investigated whether PpIX can also serve as a serum HGG marker to monitor relapse. Patients (HGG: n = 23 primary, pHGG; n = 5 recurrent, rHGG) undergoing FGR received 5-ALA following standard clinical procedure. The control group of eight healthy volunteers (HCTR) also received 5-ALA. Serum was collected before and repeatedly up to 72 h after drug administration. Significant PpIX accumulation in HGG was observed after 5-ALA administration (ANOVA: *p* = 0.005, post-hoc: HCTR *vs*. pHGG *p* = 0.029, HCTR *vs*. rHGG *p* = 0.006). Separation of HCTR from pHGG was possible when maximum serum PpIX levels were reached (CI_95%_ of t_Max_). ROC analysis of serum PpIX within CI_95%_ of t_Max_ showed successful classification of HCTR and pHGG (AUC_ROC_ 0.943, CI_95%_ 0.884–1.000, *p* < 0.001); the optimal cut-off for diagnosis was 1275 pmol PpIX/ml serum, reaching 87.0% accuracy, 90.5% positive predictive and 84.0% negative predictive value. Baseline PpIX level was similar in patient and control groups. Thus, 5-ALA is required for PpIX induction, which is safe at the standard clinical dosage. PpIX is a new target for liquid biopsy in glioma. More extensive clinical studies are required to characterize its full potential.

## Introduction

High-grade gliomas (HGG) derive from glial cells and are assigned to Grades 3 and 4 according to the World Health Organization (WHO) classification of central nerve system (CNS) tumors^1,2^. They comprise glioblastomas (GBM), IDH-mutant astrocytomas, and oligodendrogliomas (Grade 3), among others^1,2^. GBM, the most common HGG, accounts for 50% of all malignant CNS tumors^2^. Despite advances in treatment and understanding of HGG, they remain incurable^2,3^. Even after multimodal therapy, median overall survival is low (15–18 months), and the 5-year survival rate is only 7%^2,3^. GBM is more common in males than females; the overall age-adjusted incidence is 3.3 out of 100,000 individuals^2^.

Due to their rapid growth followed by displacement or infiltrative destruction of brain structures, treatment remains difficult^3^. Contrast-enhanced magnetic resonance imaging (MRI) is the gold standard for diagnosis and follow-up to monitor treatment response and tumor progression^4^. When feasible, the first-line therapy of HGG starts with surgical resection aiming for maximal tumor reduction. Current surgical adjuncts for HGG resection include, among others, neuronavigation, ultrasound, and 5-aminolevulinic acid (5-ALA)-mediated fluorescence-guided resection (FGR)^3,5^.

Initial therapy typically includes maximal resection followed by concomitant radio- and chemotherapy with temozolomide^6^. Hereafter, an MRI scan is recorded as a baseline for disease monitoring^7^. Further MRI scans for monitoring of treatment response and follow-up are regularly performed, i.e., every 3 to 6 months^7^. Thus, follow-up examinations of HGG patients come at high costs and strain; they rely on specialized centers for diagnostic imaging. Also, MRI cannot be performed in patients with metal implants or cardiac pacemakers. Despite multimodal therapy, the median interval for GBM recurrence is seven months and inevitable^3^. Once these tumors recur, median overall survival is about 6–11 months^3^. When progression is suspected with MRI, differentiation between progression and pseudoprogression is challenging. True tumor progression accounts for about 60%^3,8^ and is treated differently from pseudoprogression (10–36%) due to radiotherapy-associated changes, but not tumor regrowth^3,8^. Both forms can be differentiated by advanced MRI techniques or ^18^F-fluoroethyl-L-tyrosine positron emission tomography (^18^FET-PET). Still, verification via tissue collection during a second surgery and histopathological evaluation is often necessary^3^.

Liquid biopsies may be an early method for glioma diagnosis when tissue biopsy is not yet appropriate or feasible. They may be helpful for longitudinal disease monitoring, be specific enough to distinguish tumor progression from pseudoprogression, and may also have a prognostic and predictive value^9^. Currently, there is no validated circulating biomarker for glioma in any body fluid, despite intensive research^10^. Most attempts to find a candidate focus on circulating tumor cells (CTCs), extracellular vesicles, DNA, micro RNA, and proteins in blood or cerebrospinal fluid^9,10^.

5-ALA is an FDA-established tissue marker for HGG during FGR^11,12^ as it induces accumulation of fluorescing protoporphyrin IX (PpIX) in malignant glioma tissue. For surgery, 5-ALA is administered orally to patients four hours before induction of anesthesia^13^. The 5-ALA excess in the body circumvents the negative feedback regulation in heme biosynthesis and leads to the selective accumulation of PpIX in glioma cells^13,14^. The mechanism of this process is not entirely clear^13,15^ (for recent reviews, see^16–18^). The availability of 5-ALA from blood vessels in the brain is an essential factor^19–21^ because an intact blood–brain barrier (BBB) is impermeable to 5-ALA^13^. Thus, PpIX synthesis after exogenous 5-ALA uptake is predominantly observed in brain regions without BBB (e.g., choroid plexus) or with a disrupted BBB (HGG)^13,22,23^. Almost every cell in the human body performing cellular respiration is capable of heme biosynthesis, a pathway in which PpIX is the direct precursor of heme^14^. Heme synthesis is mainly localized in the bone marrow (erythroid precursor cells, ~ 85% of daily heme production) and the liver^14,24^.

Heme synthesis is dysregulated in cancer. For colorectal^25^, breast^26^, and kidney cancer^27^, different spectral characteristics in blood of patients or xenografts with tumor compared to a control group were reported, which were attributed to increased blood porphyrins^25–30^. The intake of 5-ALA increases this effect. Plasma PpIX was significantly elevated in patients with bladder cancer following 5-ALA administration^31^. Specifically for HGG, PpIX-containing extracellular vesicles were identified in the plasma of GBM patients three hours after 5-ALA administration (aALA) by imaging flow cytometry^32^.

Although the exact cellular mechanism for the accumulation of PpIX aALA remains diffus^13,33^, practical knowledge suggests an elevated PpIX blood level in HGG patients, reflecting the extensive PpIX accumulation in glioma tissue^13^. We hypothesize that PpIX could be a valuable blood biomarker for monitoring tumor recurrence and possibly early glioma detection.

Accurate analysis of PpIX in blood requires proper methodology. Plasma is primarily utilized for measuring porphyrin levels^25,31,34,35^. Serum has lower protein content than plasma and is closer to the in vivo conditions because no anticoagulant is required to obtain serum from whole blood. Regarding porphyrin content and pattern, both matrices, plasma, and serum, are expected to be similar. Therefore, this proof-of-concept study explores the PpIX serum kinetic in glioma patients and healthy volunteers aALA and the baseline PpIX levels without 5-ALA using a mass spectrometry (MS)-based method, which is PpIX-specific^36^.

## Methods

### Clinical procedure and sample collection

All experiments were performed in accordance with the declaration of Helsinki and with approval of the local ethics committee of the University of Münster and the Ärztekammer Westfalen-Lippe (2017–169-f-S); all patients gave informed written consent. Patients undergoing FGR with lesions suspicious for primary (n = 23, pHGG) or recurrent (n = 5, rHGG) HGG were included in the study (for details, see Table 1). Blood was collected before, during, and after surgery.

**Table 1:** Composition of the study cohort. Patient status was assessed by ECOG performance status scale and by the ASA classification system. Tissue diagnosis followed the 2021 WHO classification of CNS tumors (O^6^-methylguanine-DNA-methyltransferase (MGMT), MGMT non-methylated (MGMT ^--^), MGMT methylated (MGMT ^+^), isocitrate dehydrogenase (IDH), IDH wildtype (IDH_wt_), IDH mutant (IDH_mt_)).

Group	n	Gender		Age	ECOG		ASA		Histology	
		male	female		score	n	score	n	2021 WHO classification	n
Patients primary HGG (pHGG)	23	16 (70%)	7 (30%)	61.5 ± 13.7 (33–87)	0	13	1	3	Primary glioblastoma, MGMT ^-^, IDH_wt_	7
					1	9	2	15	Primary glioblastoma, MGMT ^+^, IDH_wt_	15
					2	1	3	5	Primary astrocytoma Grade 4, MGMT ^+^, IDH_mt_	1
Patients recurrent HGG (rHGG)	5	3 (60%)	2 (40%)	52.4 ± 19.9 (32–78)	0	1	1	-	Recurrent oligodendroglioma Grade 3, 1p/19q codeletion, IDH_mt_	1
					1	4	2	5	Recurrent glioblastoma, MGMT ^+^, IDH_wt_	3
									Recurrent glioblastoma MGMT ^-^, IDH_wt_	1
Healthy controls 5-ALA administration (HCTR)	8	4 (50%)	4 (50%)	27.6 ± 5.0 (22–35)						
Healthy controls without 5-ALA administration (HCTRw)	11	5 (46%)	6 (54%)	unknown						
Low-grade glioma (LGG)	1	1	–	18	0	1	2	1	Oligodendroglioma, Grade 2, 1p/19q codeletion, IDH_mt_	1
Radiation necrosis	1	1	–	43	1	1	2	1	Radiation necrosis and reactively altered brain tissue	1

Patients received a standard oral dose of 20 mg/kg body weight 5-ALA (Gliolan®, medac, Wedel, Germany) four hours prior to induction of anesthesia. Treatment of patients followed the standard procedures in our clinic. All patients gave informed written consent. The standard 5-ALA-dose (5-aminolevulinic acid-HCl, pharmacy, University Hospital of Münster) was also administered to a healthy control group (HCTR, n = 8). A second healthy control group without 5-ALA administration (HCTRw, n = 11) was additionally included for baseline PpIX analysis (Table 1).

Serum samples were collected according to a predefined time scheme: prior to 5-ALA administration (pALA), 0.5, 1.5, 2.5, 3.5, 4.5, 5.5, 6.0, 8.0, 10.0, 12.0 and 48 to 72 h aALA using serum-gel monovettes (S-Monovette®, Sarstedt, Nümbrecht, Germany). After centrifugation (10 min, 20 °C, 2500 rcf), serum was separated from the coagulum, aliquoted, and stored at − 20 °C until analysis. Light exposure of samples was reduced by dimming the light during blood collection, sample preparation, and analysis and by using brown sample containers and amber vials.

Data for tumor volume [cm^3^], residual tumor volume after FGR [cm^3^], body mass index (BMI), Eastern Cooperative Oncology (ECOG) performance status, classification according to the American Society of Anesthesiologists (ASA), medication and duration of anesthesia were collected, as well as clinical blood parameters (erythrocytes, leukocytes, hemoglobin, hematocrit, thrombocytes, total bilirubin, alanine-aminotransferase (ALT), aspartate-aminotransferase (AST), gamma-glutamyltransferase (gamma-GT), quick value (determined as international normalized ratio, INR), creatinine, urea) were evaluated in the context of the study. Tumor volume was calculated with commercially available software (Brainlab Elements, Brainlab AG, Munich, Germany). The data of this study is available in the supplementary data file S1.

### Protoporphyrin IX quantification in serum

**Figure 1:**
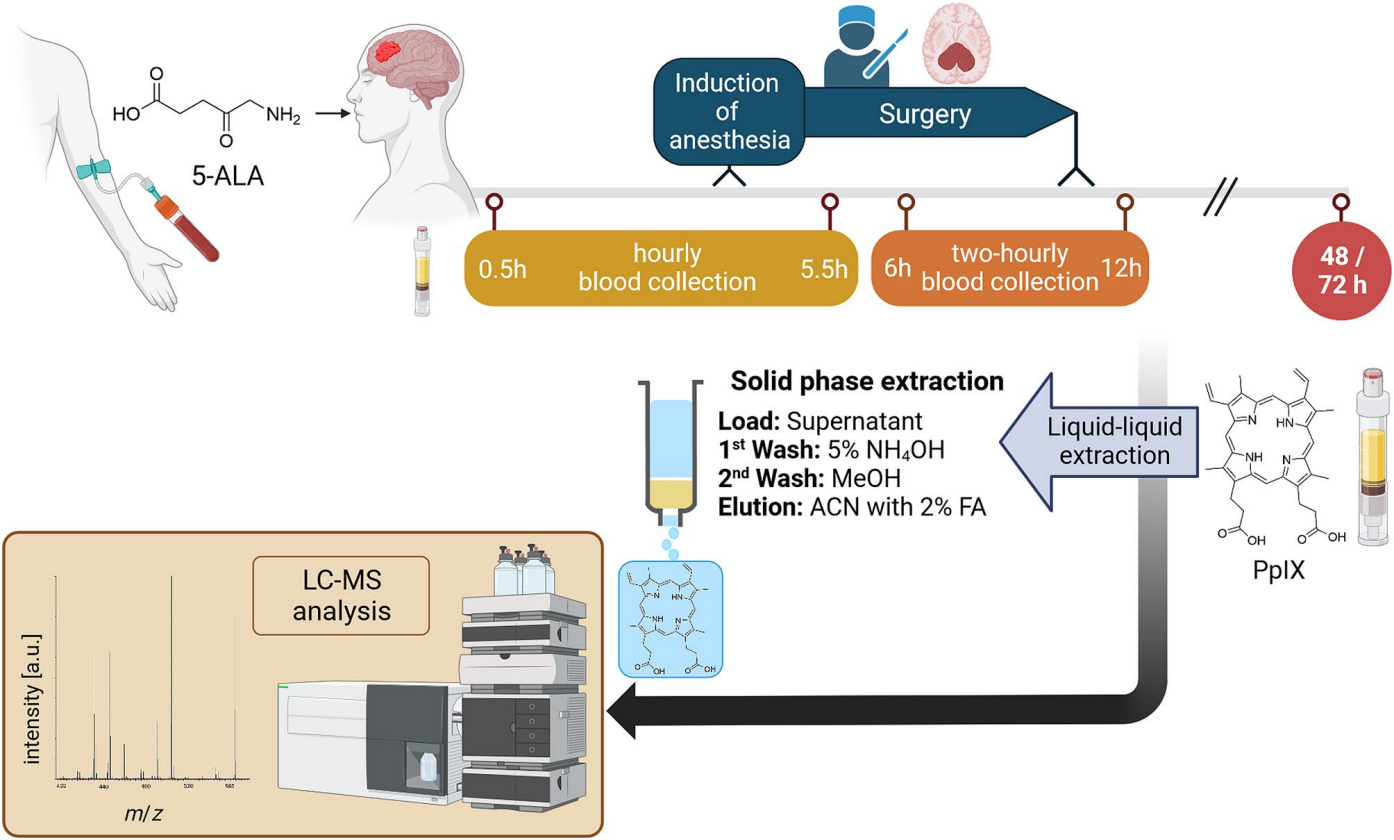
Workflow for clinical sample collection and subsequent LC–MS analysis, created with BioRender.com.

In blood samples with low expected PpIX levels (HCTRw; HCTR, pHGG, rHGG: pALA and ≥ 48 h aALA), 500 µl serum was prepared for liquid chromatography (LC)-MS analysis; otherwise (HCTR, pHGG, rHGG: 0.5–12.0 h aALA), 100 µl serum was sufficient. The internal standard mesoporphyrin IX (MpIX, Merck KgaA, Darmstadt, Germany) was added to each serum sample, yielding 0.5 pmol/µl in the final extract for analysis. Liquid–liquid extraction (LLE) of PpIX from serum was achieved by adding four parts of water and ten parts of acetonitrile (ACN) to the serum volume in two steps. Samples were shaken for one hour after adding water for hemolysis, then ACN was added, and samples were shaken again for one hour for protein precipitation and porphyrin extraction. After LLE, samples were centrifuged (30 min, 16000 rcf), and the supernatant was transferred to an anionic-exchange solid phase extraction (ae-SPE) cartridge (Oasis MAX 3cc, Waters, Eschborn, Germany). The cartridge was washed with 2 ml 5% (v/v) ammonium hydroxide solution and 2 ml methanol. Porphyrins were eluted with 2 ml ACN containing 2% formic acid. The eluate was dried using a SpeedVac concentrator (2 h, 35 °C, Savant SPD 111V with vapor trap Savant RVT 5105, Thermo Fisher Scientific, Schwerte, Germany). The residue was reconstituted in 35 µl dimethyl sulfoxide (DMSO) for samples with low expected PpIX levels (HCTRw; HCTR, pHGG, rHGG: pALA and ≥ 48 h aALA) or in 40 µl DMSO for samples taken 0.5–12.0 h aALA. Particles were removed prior to LC with centrifugal filter units (11 min, 12000 rcf, wwPTFE, pore size 2 µm, PALL Nanosep®, Dreieich, Germany). The workflow for blood sample collection and LC–MS analysis is summarized in Fig. 1.

Serum extracts were analyzed with three technical replicates using high-performance (HP)LC (HP1100, Agilent, Waldbronn, Germany) coupled to an Esquire 3000 ion trap mass spectrometer (MS, Bruker Corp., Bremen, Germany). SkyLine software (Version 21, MacCoss Lab Software) was used to evaluate HPLC–MS data^36^.

Calibration was run before each batch of 90 samples. Every calibration sample was freshly prepared using pooled serum from the 11 HCTRw. For one calibration experiment, 14 samples and two negative controls were extracted according to the protocol for high PpIX levels. Spiked PpIX concentrations were 5, 20, 100, 500, 1000, 1250, 1500, 2500, 3500, 4700, 6000, 7000, 8000, and 10000 pmol/ml serum, respectively. The MpIX concentration was 500 pmol/ml.

### Statistics

Statistical analysis was performed using SPSS software (Version 27, IBM, Germany). Data distribution was evaluated using histograms and Shapiro–Wilk test. If variables were not normally distributed, non-parametric procedures (Kruskal–Wallis with post-hoc Dunn-Bonferroni, Mann–Whitney U (MWU), Friedmann, Wilcoxon test) were used for comparison. One-way analysis of variance (ANOVA) with post-hoc Tukey test and t-tests were used if the criteria for these parametric tests were met. Spearmann-Rho correlation was additionally used for evaluation. All reported *p*-values were two-tailed. A *p*-value < 0.05 was considered statistically significant.

The area under the curve of PpIX serum levels (AUC_SumPpIX_) was calculated according to the linear method (Eq. 1) and summed up for samples taken pALA until 12 h aALA. To evaluate the potential of serum PpIX to discriminate between healthy and pHGG patients, receiver operating characteristic (ROC) analysis was performed.

Equation 1: Calculation of AUC_SumPpIX_ for PpIX formation in serum (t = time aALA[h], C = PpIX serum level [pmol/ml]).

1 $${\text{AUC}}_{{0 - {\text{t}}_{{\text{n}}} }} = \frac{1}{2} \mathop \sum \limits_{{{\text{i}} = 1}}^{{{\text{n}} - 1}} ({\text{t}}_{{{\text{i}} + 1}} - {\text{t}}_{{\text{i}}} ) ({\text{C}}_{{\text{i}}} + {\text{C}}_{{{\text{i}} + 1}} )$$

## Results and discussion

### Baseline PpIX serum levels

**Figure 2:**
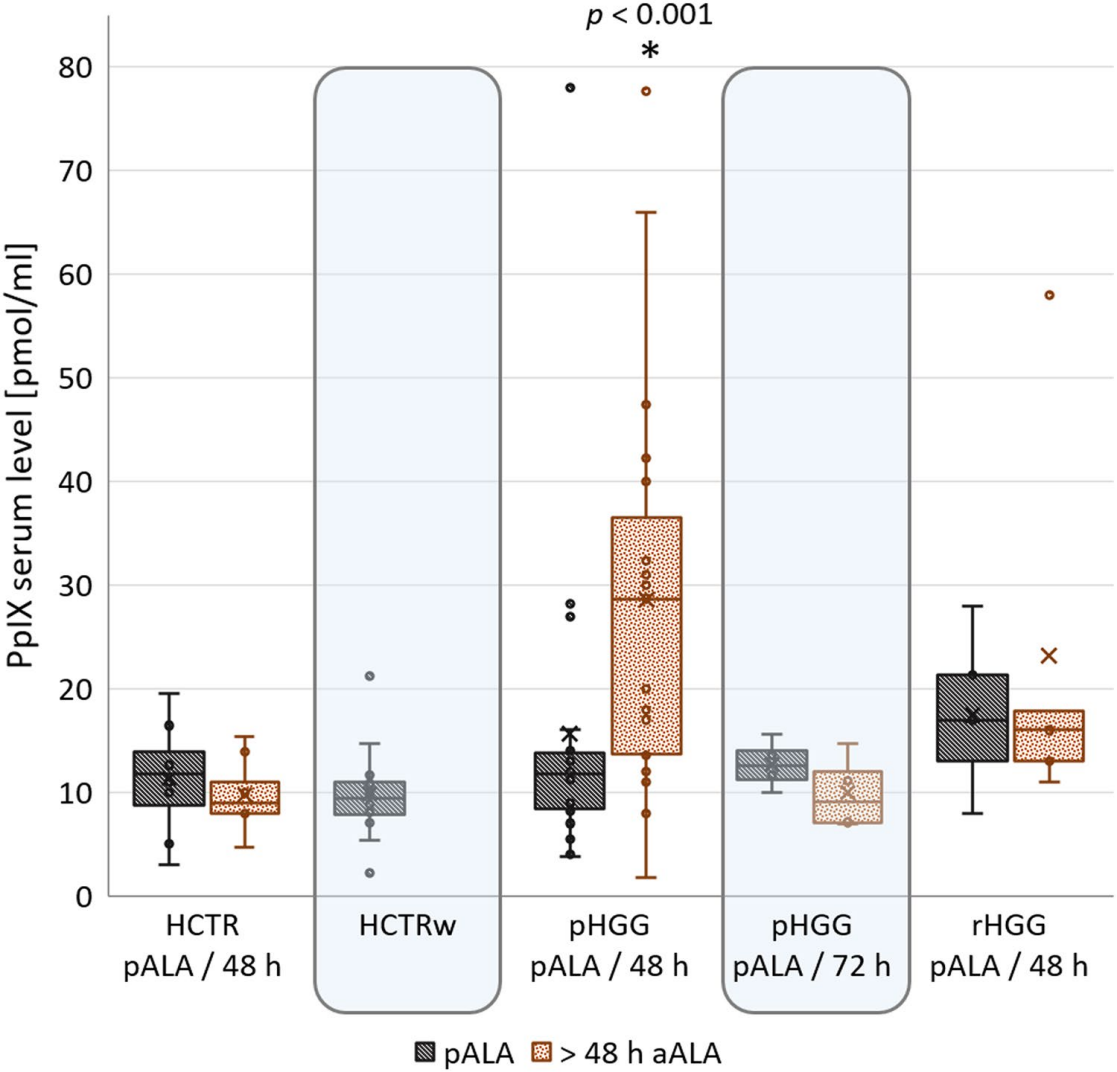
Baseline PpIX levels pALA and levels ≥ 48 h aALA. The differences in PpIX levels pALA between HCTR, HCTRw, pHGG, and rHGG were not significant (Kruskal–Wallis: *p* = 0.121). Only the PpIX level in pHGG 48 h aALA was significantly higher than the baseline in all groups (MWU: *p* < 0.001).

Endogenous PpIX levels pALA and without drug usage were low (14 ± 11, 2–78 pmol/ml (mean ± standard deviation (sd), range), CI_95%_ 10–17 pmol/ml, n = 47), and there were no significant differences among the groups (Fig. 2, black square). There is no reference range for PpIX in serum for healthy individuals, but 2–15 pmol PpIX/ml in plasma is considered normal for assessing porphyria based on HPLC-FLD analysis^34^. However, despite laborious protocols, PpIX often could not be reliably measured in the past^34^ or was not detected in plasma samples at all^35^. PpIX plasma concentrations reported in the literature ranged from below the method-dependent limits of quantification to hundreds of nmol/l^31,35^. Reported results were strongly dependent on the analysis method^36^.

In cancer patients, increased blood porphyrin levels were described without 5-ALA administration^25–30^ based on the analysis of fluorescence spectra. Thereby, an acetone extract of the residue from anticoagulated whole blood after removal of plasma was used for fluorophotometry^26–30^. Thus, the detected fluorophores originated mainly from red blood cells (RBC), not plasma. When excited in the range of the Soret-band (400, 405 nm)^26,28,29^, characteristic emission was observed at ~630 nm^26–30^. This signal was in some cases directly attributed to PpIX^28,29^ but primarily to elevated blood porphyrins in general^25,26,29,30^. Given the similar fluorescence characteristics of porphyrins^37,38^ and interferences from other intrinsic blood fluorophores (e.g., tryptophan, reduced nicotinamide adenine dinucleotide, flavins)^39,40^, individual porphyrins cannot be differentiated in blood by FLD alone. Fluorophotometry was rarely used to analyze the acetone extract of RBC and native plasma in parallel^29,30^, although their fluorescence emission profiles were highly different, which suggests that different porphyrins accumulate^29,30^.

In contrast to RBC extract, no PpIX-suspicious signal at ~630 nm was apparent in plasma^29,30^, but different porphyrins were present in both matrices. Clearly, sensitivity and specificity of fluorophotometry for PpIX detection in plasma are insufficient, leading to questions concerning the reports about elevated PpIX levels in blood of cancer patients, which were determined with this method. LC coupled to MS has been used before to characterize coproporphyrin and PpIX in plasma from colorectal adenocarcinoma patients^25^ and is an excellent means for the specific and reliable measurement and quantification of PpIX in serum.

We did not detect increased serum PpIX concentrations in HGG patients pALA by using LC–MS. Thus, to exploit the biomarker potential of PpIX in HGG, the stimulation of heme biosynthesis pathway with exogenous 5-ALA was necessary. This is in accordance with previous findings in bladder^31,41^, liver^42^, colorectal^43^, and pancreatic^44^ cancer, showing that the urinary porphyrin profile following 5-ALA administration could be a potential tumor biomarker. Differences in urinary porphyrin profiles pALA were not seen^42–44^. Additionally, in HGG, PpIX-containing extracellular vesicles were found in plasma, but only aALA and not pALA^32^.

For our study, serum was analyzed because it is more native than plasma, contains fewer proteins, and porphyrin content and pattern are similar to plasma. Serum is closer to the in vivo conditions than plasma because no anticoagulant or other supplement is required after blood collection. Whole blood contains zinc PpIX (ZnPpIX)^45^, which is rapidly hydrolyzed to free PpIX and Zn^2+^ under acidic conditions^45^. Consequently, a sum parameter of free and ZnPpIX would then be measured^36,46,47^. Moreover, ZnPpIX and free PpIX fluorescence overlaps^45,48^, which hampers reliable measurement of free PpIX next to ZnPpIX when using FLD. Serum, when properly collected and prepared, should be free of ZnPpIX.

### Serum PpIX after 5-ALA administration

Due to the human body’s capability for heme biosynthesis, increased PpIX formation was expected aALA in all groups. 5-ALA is a small (131 Da) polar molecule and rapidly absorbed after oral administration. 5-ALA plasma levels peak at 0.9 h, and the substance is quickly removed from the body with a terminal half-life of about three hours^35,49^. PpIX levels in blood also rapidly increase aALA^35,49–51^. 5-ALA supplementation seems to be well tolerated by the body^13^. Apart from the known light sensitivity of the skin 24 h aALA and rare elevation of liver enzymes, oral 5-ALA supplementation (20 mg/kg body weight) and the resulting induction of heme biosynthesis have no harmful side effects^13,52,53^.

**Figure 3:**
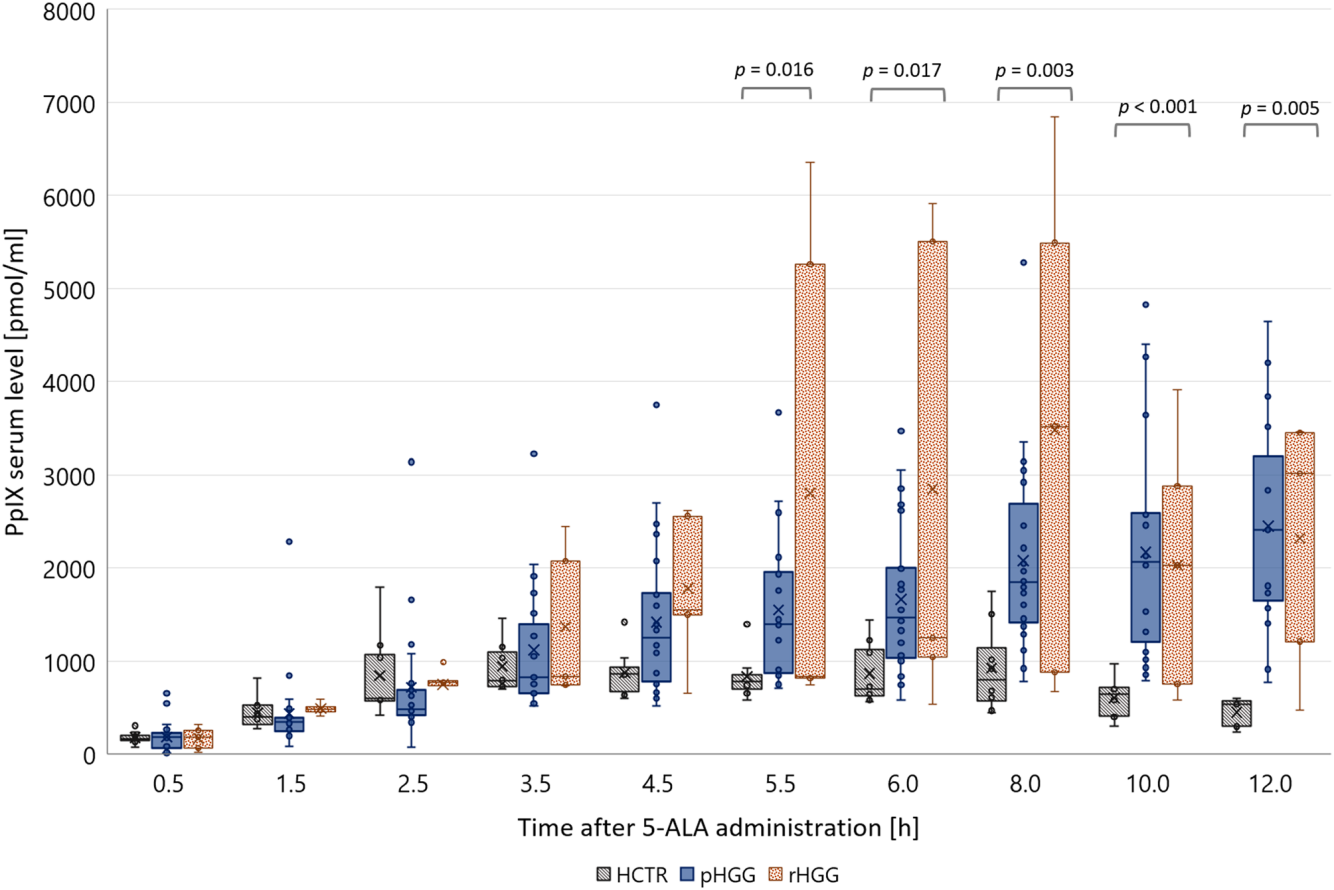
Time kinetic of PpIX formation in serum from 0.5 to 12.0 h aALA. Kruskal–Wallis with post-hoc Dunn-Bonferroni test was used to compare the groups at each time point.

In our study, the PpIX serum concentration rapidly rose aALA in all three groups (pHGG, rHGG, HCTR): 0.5 h after administration, the PpIX level was about ten-fold higher compared to that pALA. The measured values changed similarly in these groups up to 4.5 h. After that, they started to differ considerably (Fig. 3).

From 5.5 to 12.0 h aALA, PpIX serum levels were significantly elevated in HGG compared to HCTR (Kruskal–Wallis: *p* < 0.001, post-hoc Dunn-Bonferroni: HCTR *vs*. pHGG *p* < 0.001, HCTR *vs*. rHGG *p* < 0.001, pHGG *vs*. rHGG *p* = 1.000, combined samples 5.5–12.0 h aALA were compared). Statistical comparison at each time-point separately showed significant differences between pHGG and HCTR (Kruskal–Wallis test: Fig. 3, post-hoc Dunn-Bonferroni: HCTR *vs*. pHGG *p* = 0.013 (5.5 h), *p* = 0.016 (6.0 h),* p* = 0.004 (8.0 h),* p* < 0.001 (10.0 h),* p* = 0.004 (12.0 h), HCTR *vs*. rHGG and pHGG *vs*. rHGG were not significant). Most likely, this study's number of rHGG patients was too low to indicate significant differences in serum PpIX levels between HCTR and rHGG. Future studies with an increased number of patients are necessary to elaborate on whether rHGG can be discriminated from HCTR and pHGG by serum PpIX levels. Women seemed to have more PpIX than men, but this was not significant (Supplement, Figure S1). AUC of PpIX serum levels (AUC_SumPpIX_) was calculated for samples obtained pALA up to 12.0 h aALA (Eq. 1). This parameter differed significantly between HCTR and both patient groups, indicating enhanced PpIX formation in patients (ANOVA: *p* = 0.005, post-hoc Tukey test: HCTR *vs*. pHGG *p* = 0.029, HCTR *vs*. rHGG *p* = 0.006, pHGG *vs*. rHGG *p* = 0.252).

In pHGG, the PpIX level 48 h aALA was significantly ~2-fold higher than the baseline (Fig. 2, pHGG 48 h aALA *vs.* pALA: n = 19, Wilcoxon test: *p* = 0.008). In four pHGG patients, the blood sample was taken at 72 h aALA instead of 48 h aALA due to clinical procedures. In these four samples, PpIX had returned to the baseline. For HCTR and rHGG, there was no significant difference in the baseline PpIX level at 48 h aALA. In contrast, for pHGG, the 48 h level was still significantly higher than the baseline in all groups (MWU test: *p* < 0.001, Fig. 2), indicating more extended clearance in pHGG.

### Evaluation of t_Max_ and PpIX_Max_

Two essential characteristics of the measured PpIX curves were the maximum PpIX level (PpIX_Max_ [pmol/ml serum]) and the time point at which PpIX_Max_ was reached (t_Max_ [h aALA]). PpIX_Max_ was significantly higher in pHGG and rHGG than in HCTR (Kruskal–Wallis test: *p* = 0.001, post-hoc Dunn-Bonferroni: HCTR *vs*. pHGG *p* = 0.001, HCTR *vs*. rHGG *p* = 0.011, pHGG *vs*. rHGG not significant). For pHGG, PpIX_Max_ was nearly 3-fold higher compared to HCTR (2742 ± 1186, 852–5281 pmol/ml *vs*. 1032 ± 385, 649–1707 pmol/ml (mean ± sd, range)) and for rHGG, even higher values were reached (3863 ± 2671, 1044–6848 pmol/ml (mean ± sd, range)).

t_Max_ was also different in HGG compared to HCTR; in both patient groups, PpIX levels peaked later (pHGG: 8.9 ± 2.3, 4.5–12.2 h aALA; rHGG: 6.4 ± 1.6, 4.5–8.0 h aALA, HCTR: 5.0 ± 1.6, 2.5–8.0 h aALA (mean ± sd, range)). PpIX formation and/or degradation was delayed in rHGG by 1.5 to almost 4.0 h in pHGG. Additionally, t_Max_ showed high individual variance with ranges overlapping among the groups. The differences in t_Max_ were significant for comparison between HCTR and pHGG (ANOVA: *p* < 0.001, post-hoc Tukey test: *p* < 0.001); rHGG showed a delay in t_Max_, but probably due to the small number of individuals (n = 5) this was not significant. The literature reports PpIX blood maxima within a range of 5–12 h aALA and large individual variations^50,54^, but mainly PpIX peaked within 7–9 h^49,54^. PpIX fluorescence in glioma tissue, as determined in the context of research in FGR, reached the highest values 7–8 h aALA for strong and 8–9 h for weak fluorescing samples^55^ matching the values observed in the present study. Within 8–10 h aALA, PpIX tissue fluorescence decreased^55,56^. For skin, PpIX fluorescence maxima were measured between 6.5 h aALA at the back of the hand and 9.8 h at the forearm^51^.

### Correlation with clinical parameters

PpIX results (PpIX_Max_, t_Max_, AUC_SumPpIX_) were tested against tumor volume, residual tumor volume after FGR, BMI, ECOG and ASA score, duration of anesthesia [h], total of medications taken, and patient age using a non-parametric Spearmann-Rho approach. PpIX_Max_ and AUC_SumPpIX_ correlated significantly (r_s_ = 0.918, *p* < 0.001, n = 28), which is reasonable because a generally higher formation of PpIX is related to a higher PpIX_Max_. Additionally, the duration of anesthesia and t_Max_ correlated significantly (r_s_ = 0.667, *p* = 0.003, n = 19). t_Max_ occurred later with prolonged anesthesia, which slowed down 5-ALA/PpIX metabolism. It is well known that anesthetic agents affect global oxidative metabolism, cerebral blood flow, and endogenous regulatory mechanisms such as cerebral autoregulation, vasomotor reactivity, and neurovascular coupling^57^. We have also noted more extended clearance in pHGG patients. In healthy volunteers, serum PpIX returned to the baseline within 48 h aALA; at this time, it was still elevated in pHGG patients (Fig. 2).

No other correlations were found, especially no effect on PpIX_Max_ and AUC_SumPpIX_ was detected. There was also no effect of individual medication apart from an earlier t_Max_ with the intake of proton pump inhibitor Pantoprazole (intake: t_Max_ = 7.9 ± 2.3 h aALA, n = 22; no intake: t_Max_ = 10.4 ± 1.5 h aALA, n = 6, t-Test: *p* = 0.020; pairwise comparison of medication groups using MWU or t-Test dependent on data distribution). t_max_ was approximately equal in men and women, and PpIX_Max_ was slightly increased in women. For both parameters, differences were not significant between the sexes.

Hematology parameters (erythrocytes, leukocytes, hemoglobin, hematocrit, thrombocytes) were evaluated, as well as parameters for liver (total bilirubin, ALT, AST, gamma-GT, INR) and kidney (creatinine, urea). To assess blood parameters, the last blood count before surgery was considered baseline and compared to the early postoperative blood count within 24 h after surgery. Most parameters remained within the reference range; only the average for erythrocytes, hemoglobin, and hematocrit was significantly lower after the surgery compared to the baseline (Wilcoxon test: *p* < 0.001 each) and were on average lower than the reference range (erythrocytes 3.8 ± 0.4*10^6^/µl (mean ± sd), reference 3.92–5.08*10^6^/µl; hemoglobin 11.5 ± 1.3 g/dl, reference 11.9–14.6 g/dl; hematocrit 35.1 ± 11.0%, reference 36.6–44.0%).

A specific effect of the surgical procedure on PpIX formation or degradation was thus not evident. All evaluated blood parameters were within the reference range except for postoperatively lowered erythrocytes, hemoglobin, and hematocrit, probably due to the extensive volume administration during anesthesia causing blood dilution. These included liver parameters, which were particularly interesting because the liver plays a central role in heme biosynthesis and PpIX elimination^14,58^. It is assumed that PpIX is solely eliminated by hepatic clearance and secreted into the bile^14,58–60^. The first step in this mechanism, the uptake of PpIX into hepatocytes, is thought to be a first-order reaction kinetic and thus only dependent on the PpIX concentration; PpIX uptake is slow compared to other organic anions like bilirubin^60^. Details on the mechanism of hepatic clearance remain unclear, but PpIX passes the liver unchanged^61^, and intact PpIX is detected in faeces^62^. The canicular secretion is considered as rate-limiting step^60^.

### Classification of patients and healthy controls

**Figure 4:**
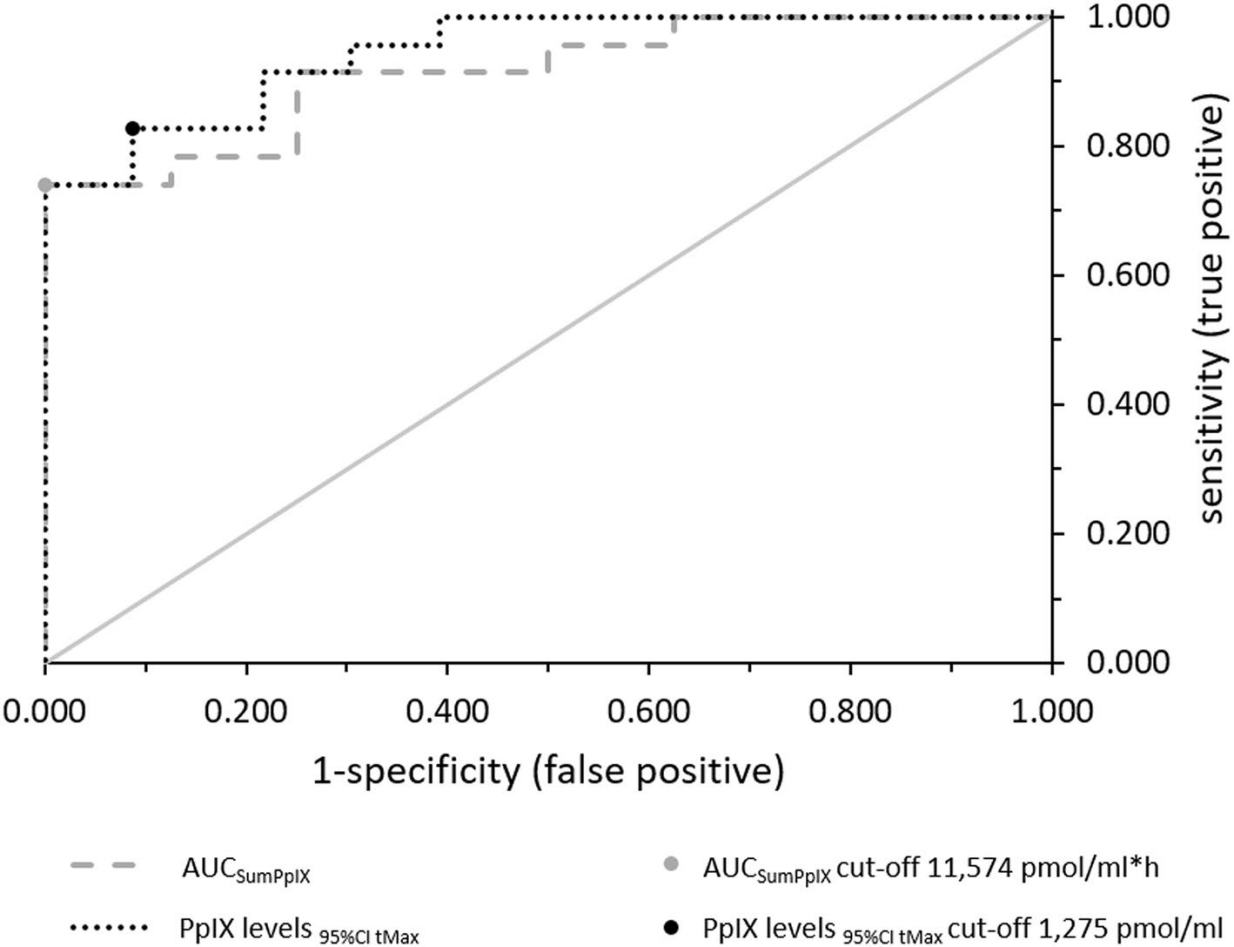
ROC analysis of AUC_SumPpIX_ and PpIX levels within CI_95%_ of t_Max_ in HCTR and pHGG. Points indicate the optimal cut-off values closest to the upper left corner; the diagonal solid line is the reference line.

ROC analysis for AUC_SumPpIX_ showed good discrimination between HCTR and pHGG (AUC_ROC_ = 0.913, *p* = 0.001, CI_95%_ = 0.813–1.000). When considering all PpIX values within the 95% confidence interval (CI_95%_) for t_Max_ of HCTR (3.7–6.4 h aALA) and pHGG (7.9–9.9 h aALA), the analysis yielded even better classification characteristics (AUC_ROC_ = 0.943, *p* < 0.001, CI_95%_ = 0.884–1.000, Fig. 4). Optimal cut-off values for diagnosing a tumorous patient against a healthy individual were 11574 pmol/ml*h for AUC_SumPpIX_ and 1275 pmol/ml for PpIX within CI_95%_ of t_Max_. With these cut-off values, test performance comparison (Table 2) showed that PpIX within CI_95%_ t_Max_ yielded better sensitivity, accuracy, and negative predictive value (NPV) than AUC_SumPpIX_. PpIX formation in HGG was higher than in HCTR until 12.0 h aALA (PpIX_Max_, AUC_SumPpIX_). Our LC–MS-based method was thus able to discriminate HGG from HCTR sera. Using PpIX levels within CI_95%_ of t_Max_, high accuracy, sensitivity, positive predictive value (PPV), and NPV were obtained.

**Table 2:** Calculated characteristics of test performance for the variables AUC_SumPpIX_ and PpIX CI_95%_ t_Max_ for discrimination of HCTR from pHGG (positive predictive value (PPV), negative predictive value (NPV)).

Variable	Specificity [%]	Sensitivity [%]	Accuracy [%]	PPV [%]	NPV [%]
AUC_SumPpIX_	100.0	73.9	80.6	100.0	57.1
PpIX CI_95%_ t_Max_	91.3	82.6	87.0	90.5	84.0

Applying the cut-off values for classification of rHGG patients (n = 5) and two additionally sampled patients (low-grade glioma (LGG) n = 1, radiation necrosis n = 1, Table 1), AUC_SumPpIX_ yielded 5/7 (71%) correct classifications. Two rHGGs were falsely classified as harboring no tumor. PpIX within CI_95%_ of t_Max_ classified 4/7 (57%) cases correctly; one rHGG was again incorrectly diagnosed as tumor-free. Two rHGG patients were inconclusive because PpIX levels within CI_95%_ of t_Max_ scattered above and below the cut-off. The radiation necrosis and LGG patients were each classified correctly with both variables. As the two patients with unclear pathologies according to the preoperative MRI (LGG, radiation necrosis) were classified correctly, these results show the potential of PpIX to assist in glioma monitoring. Further data is required to establish PpIX as a blood biomarker and evaluate its diagnostic performance in larger cohorts.

### Assessment of PpIX as HGG biomarker

Next to PpIX, other promising circulating biomarker candidates are CTCs, circulating tumor DNA (ctDNA), exosomes, micro RNA, and proteins^10^. While some of these candidates, e.g., CTCs and ctDNA, are highly specific, allowing even molecular tumor classification, they require elaborate isolation techniques and suffer from low sensitivity of tumor detection^10^. In the case of micro RNA and proteins, specificity is often lacking^10^; the link between micro RNA or protein biomarker candidates and the glioma tumor is often missing. PpIX is an established optical tissue marker during HGG resection^11–13^. Thus, PpIX is already applied in clinical routines to detect glioma tissue, and there is a relationship between PpIX as a biomarker candidate and glioma tumor. Additionally, serum PpIX determination is more robust than CTCs or ctDNA analysis and requires less laborious protocols. For instance, the LC–MS workflow, which was developed for this clinical proof-of-concept study, can easily be transferred to any laboratory with a mass spectrometer.

Although discrimination of pHGG from HCTR via serum PpIX was successful in our study, questions requiring further elucidation studies remain. For instance, the impact of the BBB permeability is unclear. BBB disruption will probably affect the sensitivity of the PpIX biomarker concept. The exact mechanism of PpIX accumulation in HGG aALA is not entirely clear yet^13,15^. Still, BBB disruption is essential for the uptake of 5-ALA in malignant glioma^13,19–21,63^.

Moreover, elevated serum PpIX may not exclusively be related to HGG aALA. Instead, porphyrins have been investigated in a variety of oncological diseases, including prostate tumors^28,64^, breast cancer^26^, renal cell carcinoma^27^, colorectal cancer^25,43^, bladder cancer^31,41^, hepatocellular carcinoma^42^ and pancreatic cancer^44^. It was extrapolated that the accumulation of porphyrins is common to almost all types of cancer^26–28^ and that the specific measurement of PpIX is advantageous for cancer screening in general^31^. Still, most studies lack evidence on whether porphyrins in general or a specific porphyrin, e.g., PpIX was elevated. Due to nonspecific analytical methodologies, porphyrin discrimination was often not achieved^25–29,65^. Moreover, lead poisoning, iron deficiency anemia, and porphyria can cause elevation of blood porphyrins^24,66^. While serum PpIX alone may lack specificity for initial glioma diagnosis, it could assist in surgical decision-making. Additionally, PpIX has the potential to aid in monitoring glioma recurrence and could possibly replace the need for repeated MRI scans with contrast agents. It could be standard practice to perform an MRI scan only after PpIX serum analysis suggests tumor progression. Furthermore, serum PpIX may help distinguish between true glioma progression and pseudoprogression, which can be challenging to discriminate using solely imaging techniques (MRI, ^18^FET-PET)^3^. Often, histopathological evaluation of biopsied tissue is required for clarification^3^.

### Limitations of the conducted study

This first proof-of-concept study included only a small number of healthy controls and patients. Thus, no separation into a discovery and validation cohort for the potential biomarker was possible. Moreover, patients were anesthetized and treated according to standard clinical procedures, while healthy controls were not. We evaluated this potential bias by analyzing clinical parameters. No specific effect on heme biosynthesis and the total amount of synthesized PpIX (PpIX_Max_, AUC_SumPpIX_) was detectable. Therefore, classification analysis was feasible, and ROC analysis yielded promising results. Since anesthesia affected the 5-ALA/PpIX metabolism by shifting PpIX maxima to later time points for potential diagnostic use, it will be necessary to determine the time kinetics of PpIX serum levels in patients unaffected by surgery.

## Conclusion

Discrimination between HGG patients and healthy controls is feasible by serum PpIX analysis following oral 5-ALA administration. We detected ~200-fold maximum serum levels compared to endogenous levels in pHGG in anesthetized patients between 4.5 and 12.2 h aALA (CI_95%_ 7.9–9.9 h aALA) undergoing FGR. This first study discovered the potential of PpIX for a novel liquid biopsy approach in HGG. A test on a single blood sample drawn within the proper timeframe could indicate tumor presence or recurrence. Ultimately, these results still need to be proven in further studies with larger cohorts and an adapted study design.

### Supplementary Information

Supplementary Information 1.

Supplementary Information 2.

## Data Availability

All generated data are made available in the Supplement.

## References

[1] Louis DN (2021). The 2021 WHO classification of tumors of the central nervous system: A summary. Neuro-Oncol..

[2] Ostrom QT (2022). CBTRUS statistical report: Primary brain and other central nervous system tumors diagnosed in the United States in 2015–2019. Neuro-Oncol..

[3] Wen PY (2020). Glioblastoma in adults: A Society for Neuro-Oncology (SNO) and European Society of Neuro-Oncology (EANO) consensus review on current management and future directions. Neuro-Oncol..

[4] Grochans S (2022). Epidemiology of glioblastoma multiforme - literature review. Cancers.

[5] Sanai N, Berger MS (2008). Glioma extent of resection and its impact on patient outcome. Neurosurgery.

[6] Stupp R (2005). Radiotherapy plus concomitant and adjuvant temozolomide for glioblastoma. N. Engl. J. Med..

[7] Weller M (2021). EANO guidelines on the diagnosis and treatment of diffuse gliomas of adulthood. Nat. Rev. Clin. Oncol..

[8] Abbasi AW (2018). Incidence of tumour progression and pseudoprogression in high-grade gliomas: A systematic review and meta-analysis. Clin. Neuroradiol..

[9] Soffietti R (2022). Liquid biopsy in gliomas: A RANO review and proposals for clinical applications. Neuro-Oncol..

[10] Jones J, Nguyen H, Drummond K, Morokoff A (2021). Circulating biomarkers for glioma: A review. Neurosurgery.

[11] Hadjipanayis CG, Stummer W (2019). 5-ALA and FDA approval for glioma surgery. J. Neurooncol..

[12] Fisher JP, Adamson DC (2021). Current FDA-approved therapies for high-grade malignant gliomas. Biomedicines.

[13] Stepp H, Stummer W (2018). 5-ALA in the management of malignant glioma. Lasers Surg. Med..

[14] Sachar M, Anderson KE, Ma X (2016). Protoporphyrin IX: The good, the bad, and the ugly. J. Pharmacol. Exp. Ther..

[15] McNicholas K, MacGregor MN, Gleadle JM (2019). In order for the light to shine so brightly, the darkness must be present—Why do cancers fluoresce with 5-aminolaevulinic acid?. Br. J. Cancer.

[16] Kiening M, Lange N (2022). A recap of heme metabolism towards understanding protoporphyrin IX selectivity in cancer cells. Int. J. Mol. Sci..

[17] Traylor JI, Pernik MN, Sternisha AC, McBrayer SK, Abdullah KG (2021). Molecular and metabolic mechanisms underlying selective 5-aminolevulinic acid-induced fluorescence in gliomas. Cancers.

[18] Lai HW, Nakayama T, Ogura S-I (2021). Key transporters leading to specific protoporphyrin IX accumulation in cancer cell following administration of aminolevulinic acid in photodynamic therapy/diagnosis. Int. J. Clin. Oncol..

[19] Mazurek M, Szczepanek D, Orzyłowska A, Rola R (2022). Analysis of factors affecting 5-ALA fluorescence intensity in visualizing glial tumor cells—Literature review. Int. J. Mol. Sci..

[20] Harmatys KM, Musso AJ, Clear KJ, Smith BD (2016). Small molecule additive enhances cell uptake of 5-aminolevulinic acid and conversion to protoporphyrin IX. Photochem. Photobiol. Sci..

[21] Hagiya Y (2012). Pivotal roles of peptide transporter PEPT1 and ATP-binding cassette (ABC) transporter ABCG2 in 5-aminolevulinic acid (ALA)-based photocytotoxicity of gastric cancer cells *in vitro*. Photodiagn. Photodyn. Ther..

[22] Olivo M, Wilson B (2004). Mapping ALA-induced PPIX fluorescence in normal brain and brain tumour using confocal fluorescence microscopy. Int. J. Oncol..

[23] Roberts DW (2011). Coregistered fluorescence-enhanced tumor resection of malignant glioma: Relationships between δ-aminolevulinic acid-induced protoporphyrin IX fluorescence, magnetic resonance imaging enhancement, and neuropathological parameters. J. Neurosurg..

[24] Balwani M, Desnick RJ (2012). The porphyrias: Advances in diagnosis and treatment. Blood.

[25] Lualdi M (2018). Early detection of colorectal adenocarcinoma: A clinical decision support tool based on plasma porphyrin accumulation and risk factors. BMC Cancer.

[26] Kalaivani R (2008). Fluorescence spectra of blood components for breast cancer diagnosis. Photomed. Laser Surg..

[27] Courrol LC (2007). Study of blood porphyrin spectral profile for diagnosis of tumor progression. J. Fluoresc..

[28] de Oliveira Silva FR (2010). Intrinsic fluorescence of protoporphyrin IX from blood samples can yield information on the growth of prostate tumours. J. Fluoresc..

[29] Masilamani V (2012). Fluorescence spectra of blood and urine for cervical cancer detection. J. Biomed. Opt..

[30] Alsalhi M (2011). Detection of cancer by optical analysis of body fluids—A single blind study. Technol. Cancer res. Treat..

[31] Ota U (2015). Plasma protoporphyrin IX following administration of 5-aminolevulinic acid as a potential tumor marker. Mol. Clin. Oncol..

[32] Jones PS (2019). Characterization of plasma-derived protoporphyrin-IX-positive extracellular vesicles following 5-ALA use in patients with malignant glioma. EBioMedicine.

[33] Ohgari Y (2011). Roles of porphyrin and iron metabolisms in the δ-aminolevulinic acid (ALA)-induced accumulation of protoporphyrin and photodamage of tumor cells. Photochem. Photobiol..

[34] Hindmarsh JT, Oliveras L, Greenway DC (1999). Plasma porphyrins in the porphyrias. Clin. Chem..

[35] Dalton JT (2002). Clinical pharmacokinetics of 5-aminolevulinic acid in healthy volunteers and patients at high risk for recurrent bladder cancer. J. Pharm. Exp. Ther..

[36] Walke A, Suero Molina E, Stummer W, König S, Mitulović G (2020). Protoporphyrin IX analysis from blood and serum in the context of neurosurgery of glioblastoma. Mass Spectrometry in Life Sciences and Clinical Laboratory.

[37] Seo I, Tseng SH, Cula GO, Bargo PR, Kollias N (2009). Fluorescence spectroscopy for endogenous porphyrins in human facial skin. Proc. SPIE Int. Soc. Opt. Eng..

[38] Plavskii VY (2018). Porphyrins and flavins as endogenous acceptors of optical radiation of blue spectral region determining photoinactivation of microbial cells. J. Photochem. Photobiol. B.

[39] Weber A, Lednev IK (2022). Brightness of blood: Review of fluorescence spectroscopy analysis of bloodstains. Front. Anal. Sci..

[40] Li B-H, Zhang Z-X, Xie S-S, Chen R (2006). Fluorescence spectral characteristics of human blood and its endogenous fluorophores. Spectrosc. Spectr. Anal..

[41] Inoue K (2013). Porphyrins as urinary biomarkers for bladder cancer after 5-aminolevulinic acid (ALA) administration: The potential of photodynamic screening for tumors. Photodiagn. Photodyn. Ther..

[42] Ishizuka M (2011). Porphyrins in urine after administration of 5-aminolevulinic acid as a potential tumor marker. Photodiagnosis Photodyn. Ther..

[43] Kamada Y (2016). Urinary 5-aminolevulinic acid concentrations as a potential tumor marker for colorectal cancer screening and recurrence. Anticancer Res..

[44] Ikeura T (2020). Effectiveness of photodynamic screening using 5-aminolevulinic acid for the diagnosis of pancreatic cancer. Anticancer Res..

[45] Labbé RF, Vreman HJ, Stevenson DK (1999). Zinc protoporphyrin: A metabolite with a mission. Clin. Chem..

[46] Piomelli S, Davidow B, Guinee VF, Young P, Gay G (1973). A micromethod for free erythrocyte porphyrins: The FEP test. J. Lab. Clin. Med..

[47] Piomelli S (1977). Free erythrocyte porphyrins in the detection of undue absorption of Pb and of Fe deficiency. Clin. Chem..

[48] Chen Q, Hirsch RE (2006). A direct and simultaneous detection of zinc protoporphyrin IX, free protoporphyrin IX, and fluorescent heme degradation product in red blood cell hemolysates. Free Radic. Res..

[49] Stummer W, Stepp H, Wiestler OD, Pichlmeier U (2017). Randomized, prospective double-blinded study comparing three different doses of 5-aminolevulinic acid for fluorescence-guided resections of malignant gliomas. Neurosurgery.

[50] Webber J, Kessel D, Fromm D (1997). Plasma levels of protoporphyrin IX in humans after oral administration of 5-aminolevulinic acid. J. Photochem. Photobiol. B.

[51] Rick K (1997). Pharmacokinetics of 5-aminolevulinic acid-induced protoporphyrin IX in skin and blood. J. Photochem. Photobiol. B.

[52] Suero Molina E, Schipmann S, Stummer W (2019). Maximizing safe resections: The roles of 5-aminolevulinic acid and intraoperative MR imaging in glioma surgery—Review of the literature. Neurosurg. Rev..

[53] Teixidor P (2016). Safety and efficacy of 5-aminolevulinic acid for high-grade glioma in usual clinical practice: A prospective cohort study. PloS One.

[54] Webber J, Kessel D, Fromm D (1997). Side effects and photosensitization of human tissues after aminolevulinic acid. J. Surg. Res..

[55] Kaneko S, Suero Molina E, Ewelt C, Warneke N, Stummer W (2019). Fluorescence-based measurement of real-time kinetics of protoporphyrin IX after 5-aminolevulinic acid administration in human *in situ* malignant gliomas. Neurosurgery.

[56] Stummer W (1998). *In vitro* and *in vivo* porphyrin accumulation by C6 glioma cells after exposure to 5-aminolevulinic acid. J. Photochem. Photobiol. B.

[57] Slupe AM, Kirsch JR (2018). Effects of anesthesia on cerebral blood flow, metabolism, and neuroprotection. J. Cereb. Blood Flow Metab..

[58] Bloomer JR (1998). Liver metabolism of porphyrins and haem. J. Gastroenterol. Hepatol..

[59] Bloomer JR (1988). The liver in protoporphyria. Hepatology.

[60] Avner DL, Berenson MM (1982). Hepatic clearance and biliary secretion of protoporphyrin in the isolated, *in situ*-perfused rat liver. J. Lab. Clin. Med..

[61] Cox TM, Alexander GJ, Sarkany RP (1998). Protoporphyria. Semin. Liver Dis..

[62] Di Pierro E (2021). Laboratory diagnosis of porphyria. Diagnostics.

[63] Suero Molina E, Black D, Kaneko S, Müther M, Stummer W (2022). Double dose of 5-aminolevulinic acid and its effect on protoporphyrin IX accumulation in low-grade glioma. J. Neurosurg..

[64] Gotardelo DR, Courrol LC, Bellini MH, de Oliveira Silva FR, Soares CRJ (2018). Porphyrins are increased in the faeces of patients with prostate cancer: A case-control study. BMC Cancer.

[65] Lualdi M (2007). Natural fluorescence spectroscopy of human blood plasma in the diagnosis of colorectal cancer: Feasibility study and preliminary results. Tumori.

[66] Lamola AA, Yamane T (1974). Zinc protoporphyrin in the erythrocytes of patients with lead intoxication and iron deficiency anemia. Science.

