# Supplementary material for: Protoporphyrin IX in serum of high-grade glioma patients: A novel target for disease monitoring via liquid biopsy
Source: Sci Rep. 2024 Feb 21;14:4297. doi: 10.1038/s41598-024-54478-y (PMC10881484; doi:10.1038/s41598-024-54478-y)
Supplement: Supplementary file 1 — Supplementary Information 1. [file 41598_2024_54478_MOESM1_ESM.docx]

# Supplement

# Protoporphyrin IX in serum of high-grade glioma patients: A novel target for disease monitoring *via* liquid biopsy

Anna Walke^1,2,^*, Christopher Krone^1^, Walter Stummer^1^, Simone König^2,+^ and Eric Suero Molina^1,+,^*

^+^shared senior authorship

*corresponding authors

^1^Department of Neurosurgery, University Hospital of Münster, Münster, Germany.

^2^Core Unit Proteomics, Interdisciplinary Centre for Clinical Research, University of Münster, Münster, Germany.


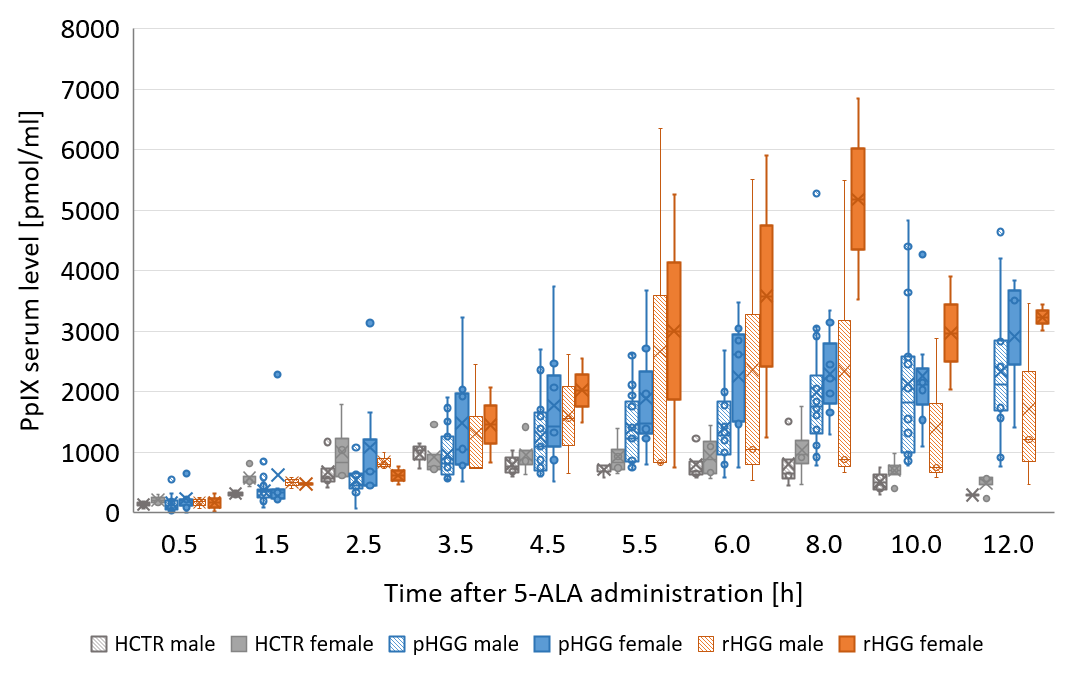


Figure S1: Time kinetic of PpIX formation in serum from 0.5 to 12.0 h aALA separated in male and female individuals within the groups of HCTR, pHGG, and rHGG. Women seemed to have higher serum PpIX levels than men, but this was not significant (Mann Whitney U test, each time point separately).
